# “After viral load testing, I get my results so I get to know which path my life is taking me”: qualitative insights on routine centralized and point-of-care viral load testing in western Kenya from the Opt4Kids and Opt4Mamas studies

**DOI:** 10.1186/s12913-022-08593-z

**Published:** 2022-12-17

**Authors:** Shirley Rui W. Qian, Shukri A. Hassan, Andrea J. Scallon, Patrick Oyaro, Evelyn Brown, James Wagude, Irene Mukui, Eunice Kinywa, Frederick Oluoch, Francesca Odhiambo, Boaz Oyaro, Leonard Kingwara, Nashon Yongo, Enericah Karauki, Jody Gao, Lindah Otieno, Grace C. John-Stewart, Lisa L. Abuogi, Rena C. Patel

**Affiliations:** 1 School of Public Health, University of Washington, Seattle, WA USA; 2 Department of Medicine, University of Washington, Seattle, WA USA; 3 Jackson School of International Studies, University of Washington, Seattle, WA USA; 4 Health Innovations Kenya (HIK), Kisumu, Kenya; 5 University of Washington-Kenya, Nairobi, Kenya; 6 Department of Health, Siaya, Kenya; 7 Drugs for Neglected Diseases Initiative, Nairobi, Kenya; 8 Department of Health, Kisumu, Kenya; 9 Family AIDS Care and Education Services, Kenya Medical Research Institute, Kisumu, Kenya; 10 Kenya Medical Research Institute-CDC, Kisian, Kenya; 11 National HIV Reference Laboratory, Kenya Ministry of Health, Nairobi, Kenya; 12 Department of Global Health, University of Washington, Seattle, WA USA; 13 Department of Pediatrics, University of Washington, Seattle, WA USA; 14 Department of Epidemiology, University of Washington, Seattle, WA USA; 15 Department of Pediatrics, University of Colorado, Denver, CO USA

**Keywords:** Viral suppression, Viral load testing, Point-of-care, Children, Pregnant/postpartum women, Kenya

## Abstract

**Background:**

Viral suppression (VS) is a marker of effective HIV therapy, and viral load (VL) testing is critical for treatment monitoring, especially in high-risk groups such as children and pregnant/postpartum women. Although routine VL testing, via centralized laboratory networks, was implemented in Kenya starting in 2014, optimization and sustainable scale up of VL testing are still needed.

**Methods:**

We conducted a mixed methods study to evaluate the impact of higher frequency, point-of-care (POC) VL testing in optimizing VS among children and pregnant/postpartum women on antiretroviral treatment (ART) in five HIV treatment facilities in western Kenya in the Opt4Kids and Opt4Mamas studies. We conducted 68 key informant interviews (KIIs) from December 2019 to December 2020 with children and pregnant women living with HIV, child caregivers, providers, laboratory/facility leadership, and county- or national-level policymakers. Our KII guide covered the following domains: (1) barriers and facilitators to ART use and VS, (2) literacy and experiences with VL in routine care and via study, and (3) opinions on how to scale up VL testing for optimal programmatic use. We used inductive coding and thematic analysis to identify dominant themes with convergent and divergent subthemes.

**Results:**

Three main themes regarding VL testing emerged from our analysis. (1) Key informants uniformly contrasted POC VL testing’s faster results turnaround, higher accessibility, and likely cost-effectiveness against centralized VL testing. (2) Key informants also identified areas of improvement for POC VL testing in Kenya, such as quality control, human resource and infrastructure capacity, supply chain management, and integration of VL testing systems. (3) To enable successful scale-up of VL testing, key informants proposed expanding the POC VL testing scheme, electronic medical records systems, conducting quality checks locally, capacity building and developing strong partnerships between key stakeholders.

**Conclusion:**

The more accessible, decentralized model of POC VL testing was deemed capable of overcoming critical challenges associated with centralized VL testing and was considered highly desirable for optimizing VS for children and pregnant/postpartum women living with HIV. While POC VL testing has the potential to improve VS rates among these populations, additional research is needed to develop strategies for ensuring the sustainability of POC VL testing programs.

**Trial registration:**

NCT03820323, 29/01/2019

**Supplementary Information:**

The online version contains supplementary material available at 10.1186/s12913-022-08593-z.

## Background

Viral suppression (VS) is a critical component of effective HIV therapy, and its inverse, viral failure, is associated with poor HIV treatment outcomes, including mortality [1, 2]. While many populations living with HIV, including general adult populations, are close to achieving the last target of VS in the UNAIDS 95–95-95 goals, some key populations, including children and pregnant/postpartum women, struggle with VS [3]. In Kenya, a high HIV-burden country, an estimated 111,500 children ages 0–14 are living with HIV [4, 5]. Only an estimated two-thirds of these children who have been on antiretroviral treatment (ART) for more than six months are virally suppressed [6]. Similarly, pregnant/postpartum women also do not always achieve or sustain VS, with 22–30% of women experiencing at least one episode of viral load (VL) ≥ 1000 copies/mL, the current threshold for viral failure according to Kenya guidelines, during pregnancy or the first six months postpartum [7–9]. The overall MTCT rates in Kenya have increased from 8% in 2016 to 11% in 2020, in part reflecting suboptimal levels of maternal VS [10, 11].

The ability to ascertain VS through frequent and easily accessible VL testing is key in treatment monitoring, however, the benefits received from current testing options are diminished by operational barriers. Beginning in 2013, the World Health Organization (WHO) advised that individuals on ART be monitored with routine VL testing [12]. Starting in 2014, Kenya adopted these recommendations and has expanded access to routine VL testing via centralized-laboratory networks, also known as centralized VL testing [13, 14]. Several potential barriers exist to centralized VL testing. Long turnaround times for test results, limited trained laboratory staff or testing expertise, difficulties in transporting samples to centralized laboratories, and the inability to monitor VL more frequently than national guidelines limit the potential benefits of centralized VL testing [8, 15–20].

Point-of-care (POC), or even near-POC, VL evaluations are feasible, accurate, and at times, less expensive than laboratory-based VL tests [21, 22]. In 2021, the WHO proposed new guidelines for POC testing engagement in HIV diagnosis and treatment and discerned several gaps in research. A strong recommendation was proposed for the use of POC HIV testing in early infant diagnosis [23]. This is supported by rich literature that demonstrated the competency of POC testing in facilitating earlier treatment initiation, higher treatment retention, and greater feasibility for resource-constrained environments [24–27]. However, the evidence base is still lacking for the effectiveness and feasibility of POC VL testing in supporting HIV treatment monitoring, including for key populations living with HIV, such as children and pregnant/postpartum women [23]. Evidence for patient, provider, policymaker perspectives on POC VL testing, including for approaches for optimization and sustained delivery of POC VL testing is also needed.

To help understand the potential impact POC, or near POC, VL testing may have on VS among the key populations, we conducted two parallel, mixed methods studies in Kenya by employing existing GeneXpert® technology implemented for POC tuberculosis (TB) testing. The first study is an open-label randomized controlled trial among children (Opt4Kids), and the second is a cohort study among pregnant/postpartum women (Opt4Mamas). Here we present our qualitative study findings that focus on patient, provider, and policymaker perspectives on how to achieve optimized and sustainable centralized and POC VL testing processes in Kenya.

## Methods

### Study setting and positionality

The study was conducted in Kisumu County, in western Kenya. Adult HIV prevalence in Kisumu is 19.9% as of 2016, which is three times higher than the national figure, while pediatric HIV incidence is the second highest [13]. Five high volume and high-HIV burden primary healthcare facilities in the Kisumu County participated in this study. Since several members of the project team were not of Kenyan background, our team took steps at study conception, design, implementation, data analysis and interpretation of findings to ensure Kenyan colleagues were involved or led aspects of this study. We conducted ad hoc meetings with the project team, which included academic researchers (including one primary investigator who belonged to the Kenyan community, one who belonged to the South Asian American immigrant community, and one who belonged to the white American community), study staff (including three who belonged to the Kenyan community, one who belonged to the Somali American immigrant community, and three who belonged to the Asian American/Pacific Islander community), and other stakeholders as needed to review our ongoing analysis and findings. The non-Kenyan members vetted the emerging themes and subthemes with our Kenyan team members to ensure the findings and interpretation resonated with their perceptions, beliefs, or lived experiences.

### Parent studies and procedures

Opt4Kids was an open-label trial that examined the impact of POC VL testing in combination with targeted drug resistance (DRM) testing and clinical decision guidance on VS among children living with HIV (CLHIV) on ART [22, 28]. The study recruited 704 children aged 1–14 years in five high-volume HIV treatment centers in Kisumu, Kenya, and followed them for the next 12 months. Eligible children were randomly assigned to undergo the intervention or to receive standard-of-care (SOC) testing. As a part of the intervention, we employed GeneXpert® technology for POC VL testing every three months, with study visits and testing schedules synchronized to intervention participants’ routine clinical visits when feasible. Study staff delivered results of POC VL testing via text messaging to participants’ primary caregiver and paper copies to providers within one to two days after testing. If lab results indicate non-suppression, the caregivers and participants were encouraged to follow-up directly with the routine providers for next steps. During the study period, samples collected for POC VL testing were centrifuged and analyzed at the onsite laboratories for four of the five study facilities equipped with the GeneXpert® platform. For the one site without GeneXpert®, samples were centrifuged at the facility laboratory and then transferred daily to a nearby study facility for GeneXpert® analysis. We administered targeted DRM testing for intervention participants with VL more than 1000 copies/mL, and results were shared with participants and their primary caregivers by their routine care providers. SOC VL testing was performed every six months and results returned through routine care, while DRM testing was limited to second-line ART failures through a centralized approval procedure in accordance with Kenyan national guidelines. The primary aim for Opt4Kids study was to assess the effect of the intervention on VS 12 months after study enrollment.

Opt4Mamas was a prospective, mixed-methods, parallel cohort study that investigated VS rates pre- and post-implementation of a POC VL testing intervention among pregnant/postpartum women living with HIV newly initiating or already on ART in the same five facilities as Opt4Kids. We enrolled 820 pregnant women during their antenatal care (ANC) visits and followed them 6 months pregnant/postpartum. Pre-intervention implementation, all enrolled women were given standard-of-care testing throughout their pregnancy/postpartum care. Post-intervention, a new cohort of pregnant women were enrolled to receive POC VL and targeted DRM testing, parallel to procedures described for Opt4Kids.

### Study procedures for qualitative data collection

For the qualitative data collection for both studies, we conducted semi-structured in-depth interviews from December 2019 to December 2020 with six subgroups of key informants, including: (1) adolescent (ages 13 and above) study participants for Opt4Kids, (2) caregivers of children enrolled in Opt4Kids, (3) women newly initiating ART during pregnancy in Opt4Mamas, (4) women already on ART with viremia at some point after enrollment in ANC in Opt4Mamas, (5) providers and other facility staff at our study sites, and (6) policymakers and other stakeholders at the local and national levels. Within the last two groups, we aimed to ensure we sampled some individuals who worked in clinical care as well as others who worked in the laboratory sections, to achieve a wide breadth of perspectives. We used convenience sampling to recruit participants/caregivers already enrolled in the parent studies who came to the clinic during our interviewing period. Additionally, we used purposive sampling to recruit providers, other facility staff, policymakers, and other stakeholders. Within each subgroup, we aimed to perform approximately 10–15 key informant interviews (KIIs), until saturation of themes was reached.

Two, related version of interview guides were used in this study. One puts more emphasis on experiences with POC VL testing, and was targeted for study participants, while the other focused on the health system perspectives of POC VL testing implementation, and was targeted for providers and policymakers. Both interview guides were designed using a socioecological model of VS, which takes into account individual, interpersonal, organizational, and structural/policy variables affecting VS, with an additional emphasis on operational aspects of POC VL and DRM testing [29]. The interview guides covered the following domains: (1) barriers and facilitators to ART usage and VS, (2) VL literacy and experiences with SOC VL and DRM testing in routine care, (3) experiences with POC VL & DRM testing via our study, and (4) how to scale up both SOC and POC VL testing for programmatic use. We specifically inquired about logistical aspects of efficiently operationalizing POC VL testing, such as how participants/caregivers like to receive results, the content and methods of results counseling, provider reaction to results receipt methods, and additional capacity-building needs for providers and health facilities. For more details, see Supplementary Files 1 and 2.

### Data collection

We obtained written informed consent from all participants in the qualitative portion of the studies, regardless of their consent for the parent studies. Adolescent participants age 13 or older also provided additional assent. We collected de-identified socio-demographic information for key informants not already participating in our parent studies on a paper form, and later entered the information into a database.

Our Kenyan study team members, who were research nurses or clinical officers, conducted the interviews and received in-person training in interviewing techniques from one of the investigators. Of note, most of the facility staff, leadership, policymakers, and other stakeholder interviews were conducted by our team’s Kenyan research coordinator. Each interview was performed in the participants' chosen language, which was either English (largely for providers and policymakers), Kiswahili, or Dholuo. The interviews were audio-recorded, and either the same interviewer or another member of the research staff transcribed the interviews directly into English if they occurred in Kiswahili or Dholuo. If the interviewer was not transcribing the recording personally, they reviewed the English transcription for accuracy and any discrepancies were resolved by discussion among research staff members. Initial transcripts were read by a principal investigator and a study coordinator, and feedbacks were provided to each interviewer for iterative improvements in interviewing as well in the guides. English transcripts were then uploaded into NVivo (version 12.0, QRS International Pty Ltd.) for coding and analysis.

### Data analysis

We used inductive coding [29, 30], and three team members carried out the coding under the supervision of a principal investigator via weekly meetings and with iterative input from the larger research team, which included members from Kenya, via as needed conference calls. One research coordinator created an initial codebook based on KII guides and her read of the initial few transcripts, and then two research assistants iteratively modified the codebook as transcript coding progressed under the supervision of the research coordinator. Initially, two transcripts were coded collaboratively, in a group setting at the same time by all three coders, and then 1–2 transcripts were double-coded separately by coders, with any differences in coding addressed through consensus. The remaining transcripts were coded independently by various coders, with one research coordinator reviewing all transcripts' coding in NVivo. We utilized thematic analysis to maintain an analytic codebook which organized our codes into overarching domains with subsequent themes, subthemes, and illustrative quotes, both convergent and divergent.

## Results

A total of 68 interviews were conducted among 63 key informants (noting 5 interviews were repeat interviews with pregnant women, one each at the start and end of study follow-up). 44 key informants were participants/caregivers (*n* = 8 with adolescent participants, *n* = 16 with caregivers of child participants, *n* = 10 with pregnant women newly initiating ART, and *n* = 10 with pregnant women with a high VL result at some point during study follow-up), 11 were providers (*n* = 6 with HIV providers or laboratory staff, *n* = 5 with facility-level leadership), and 8 were policymakers (county- or national-level policymakers). Additional demographic details of the key informants are detailed in Table 1.

**Table 1:** Key informant demographics

Frequency(*N* = 63)		
**Participants/Caregivers (*N* = 44)**		
Adolescent participants (*N* = 8)		
Age	Median	13(13–13)
Sex	Female	5
	Male	3
Caregivers of child participants (*N* = 16)		
Age of child	Median	8.5(5–11)
Sex of child	Female	5
	Male	11
Pregnant women newly initiating ART (*N* = 10)		
Age	Median	24.5(20–33)
Number of biological children	Median	1(0–2)
HIV positive children?	Yes	0
	No	10
Pregnant women with high VL during study follow-up (*N* = 10)		
Age	Median	28(24–30)
Number of biological children	Median	2(2–3)
HIV positive children?	Yes	0
	No	10
**HIV providers (*N* = 11)**		
Years of experience caring for children and pregnant women living with HIV	Median	7(3–15) years
**Policymakers (*N* = 8)**		
Years of experience caring for children and pregnant women living with HIV	Median	11(5–16) years

Below, we explore the themes related to VL testing in western Kenya from the perspectives of three large key informant groups; (1) the “patient/caregiver”, which includes study participants and caregivers for the children interviewed, (2) “providers” which includes both clinical and laboratory staff and facility leadership; and (3) “policymakers” which includes county- and national-level policymakers. In analyzing the KIIs, we comparatively investigated the emerging themes by the three groups and discuss below the dominant themes of: (1) perceived positive impacts of POC VL testing intervention; (2) perceived challenges with POC VL testing intervention; and (3) suggested areas of improvement for VL testing scale-up. Table 2 delineates the supporting quotes for these themes and relevant subthemes.

**Table 2:** Theme and quotes for the perceived impacts and areas of improvement for POC VL testing scale-up

**Main theme**	**Subtheme**	**Description**	**Supporting Quote**
Perceived positive impacts of POC VL testing intervention.	Rapid return of results or turnaround improves caretaking and facilitated timely interventions.	Enabled caregivers to reflect on their caregiving plan for their children.	*“The positive thing [of POC VL testing] is that; for example, if you take my child’s viral load and give me the results after 3 days, it will make me start thinking where I might have gone wrong so that I can improve it. And by the time I will be returning for the next visit, I will know if it is high or low. It really helps because I will do something different early enough to improve it.” (39 years old, female, caregiver)*
		Allowed providers to make timely decisions regarding patient’s treatment plan.	*“What is evident is that the turnaround time is reduced significantly; so, decisions can be made instantly…if this is made available then we can make a decision in a good time.” (Medical superintendent)*
	Decentralized POC VL testing reduces cost of test and can be used to support hard-to-reach areas.	Cost reduction by eliminating the need for transportation services.	***“****It being point-of-care means you don’t have to worry about carrying the sample from point A to point B getting a vehicle, courier services and other things…So that again becomes another beautiful thing and so it impacts on cost somehow.” (Technical advisor)*
		Accessible in hard-to-reach areas.	*“So, the introduction of point-of-care was agreed upon in the technical working group that let’s use it for the far to reach areas. In our country, there are far or hard-to-reach areas where there are delays on transportation take many hours.” (Lab director)*
	Increased testing frequency improves VL monitoring of children and pregnant/postpartum women.	Early detection of viral failure for children.	*“Because there is this norm of caregivers being changed; a child is taken to a grandmother, a child is taken to the sister or the auntie so you get adherence issue especially swallowing of antiretrovirals. So, it is better if we do it [VL testing] within three months, you can identify whether this child is failing or not.” (Nursing officer)*
		Ensure viral suppression for expectant mothers to prevent MTCT.	*“I think on a personal level like I said, it’s [three-month testing] something that even I would desire. It’s just that the national algorithm is what limits us. But it’s something to be desired when you are looking at a child or a woman, you know, pregnancy is time-bound and you want to make sure this mother is suppressed by the time they are giving birth to actually reduce the chance of transmission.” (Technical advisor)*
Perceived challenges with POC VL testing intervention.	Acceptability of POC VL testing in the healthcare sector.	Discrepancies between POC and centralized VL testing raise credibility concerns among patients.	*“Although, the viral load test results have been varying for instance, the viral load test here at the study will differ from the viral load test result from the clinic. So, you end up asking yourself if the tests are different. So, it raises a lot of questions.” (39 years old, female, caregiver)*
		Providers hope for validation and verifications of POC VL testing.	*“Now, the question of quality comes in and you know quality of testing. And you see now, from where I stand, when the question of quality comes in then you want to lean on to what you feel has been validated or what the national program or what the donor is advocating.” (Technical advisor)*
	Absence of supply chain to support POC diagnostics.	Lack of established supply chain to purchase reagents for the POC testing machines.	***“****The machine is…like right now, for example the point-of-care EID [early infant diagnosis] machine at another facility, it is not functional because it was brought under partner that their contract ended, nobody has ever bothered to buy the reagents, so the country says they do not have those cartridges in store, we have to buy them I don’t know from where and everybody feels that it is expensive, so it doesn’t help us much.” (Lab manager)*
	Capacity limitations.	Patient flow increase caused by reduction of testing turnaround time may increase workload for staff.	*“Turnaround time[of POC VL testing] has reduced so the number of patients to be tested at a given time goes up. The testing intervals is reduced, so many patients are seen at a short time; which means the staff have more workload, isn’t it? So, the staff has to cover more work.” (Medical superintendent)*
		Leverage POC testing technology for other infectious diseases testing could be met by challenges with prioritization.	*“We anticipate that since we are rolling out the multi-disease testing for VL and TB, they are also supposed to be layered on to support COVID testing in our respective counties. Yet when we reach there under multi-disease testing, there might be issues to deal with prioritization that might end up impacting the VL testing on the same equipment.” (Lab specialist)*
	Integration of POC VL testing into the current testing guidelines.	The presence of multiple testing guidance led to confusion among providers during their clinical decision-making process.	*“Because you see like right now the guidance that has been loudly spoken of is the guidance [annual or six-month testing] we’ve received from CDC, who is our primary donor that; follow the national testing platform. So now again that brings conflicts because…from what the national test results may be done less frequently than what you are seeing in the [Opt4Kids and Opt4Mamas] patient, they are three different stories then it brings a little bit of confusion…In my opinion if those things could have been sorted out and that process of integration was more seem less, then there will be have no issue, there will be no negative aspect of POC because we want POC. We want that [three-months] frequency.” (Technical advisor)*
	Anxiety towards blood draws discouraged patients from VL testing.	Children fear the painful sensation brought by blood draws.	*“That blood, when the syringe enters the veins, that is the thing I don’t like. It’s really hurting a lot.” (14 years old, male, child participant)*
		Frequent blood drawing cause anxiety among patients.	*“Some won’t come and you know pricking a client every month won’t sound so good to them. Some fear it; yes of course and some tend to think that we are collecting their blood for some other reasons… I don’t know to them what they always think but they are ever complaining that we are collecting their blood every now and then. We have to give reasons why we are doing so and we always don’t have a reason.” (Nursing officer)*
Suggested areas of improvement for VL testing scale-up.	Expansion of the POC VL testing scheme.	Policymakers hope for continual and expanded access to POC VL testing.	*"I only strongly want to recommend the rolling out of opt [Opt4Kids and Opt4Mamas] to all our health facilities…so that everyone benefits from this and they appreciate the timely ART interventions" (County-level public health authority)*
	Optimizing clinical management, workload, and utilization of POC diagnostics.	Implementing a robust health information system can drastically improve clinical management and reduce workload.	*“It’s [VL results communication] more or less, it’s running up on a paper-based system. Now, the ideal is that all the patient data is on electronic format…then the patient can actually be seen back by the clinician on the same day using a system which is available on the EMR [electronic medical records] and then the national program can also be able to get data actively within such a system…They[providers] also have so much on their hands. Especially if you have a data system that have duplicity in nature. The same data have to be entered at multiple points, then the users of the data who enters the data at times doesn’t see the value of putting in that data. So, we have a lot of data losses across of the cascade there. So, I think one of the main things is to ensure that a system is established and I think we are actually trying to support that. A system is established to avoid duplicity of data entry at various points." (Lab director)*
		Conducting local validation and quality checks for POC VL testing to build confidence among providers and other policymakers.	*“Then what are the quality issues, this is my key problem, what are the quality issues because I really want to believe that before I bring the point-of-care machine, let us run the quality checks and validation so that we can compare the results.” (Lab manager)*
	Capacity building and partnership development for sustainable VL testing system.	Integrating POC VL testing guidelines with the current national recommendation through collaborative partnership between key stakeholders.	*“Collaborating with the current implementing partner so that we harmonize the recommendations because after we get the recommendations, we have again share with the implementing partner on the ground and then we have to wait again for their views… So, if we harmonize that from our top management, it can be good and we can always deliver the services timely to the clients.” (Nursing officer)*
		Hiring more staff and buying machines to increase the capacity for POC VL testing.	***“****We need to equip the labs with POC machines and having sufficient staff to support the process.” (HIV advisor)*
	Envisioning the ideal testing experience.	Three-month testing is the ideal frequency as it allows early detection and intervention for high VL patients.	*“And after every three months, we can help those people who are failing to make a decision as fast as possible…One year [testing interval] is a long time. There are people who can be suppressed today and when you tell them your VL is suppressed and you are taking your drugs well, after that he goes and relaxes. So, by the end of one year, you might find that this patient has a lot of VL.” (Clinic manager)*
		Patients find the amount of blood draw acceptable after receiving education from providers and healthcare staff.	*“That quantity is okay… We were told that when a small amount is taken, no results can be obtained and so they must work with a certain quantity to get the required result…If you don’t know anything, you should ask. In the past, we had concerns but it was explained to us and now we know.” (36 years old, female, caregiver)*
		Performing POC VL testing in the laboratory is preferred; testing at lab can minimize contamination and improve clinical flow.	*“Lab is okay because you wouldn’t have to mess up within because everything is arranged well; the syringes and everything else but a room like this, when you want to take the sample then someone knocks at the door. At the lab, people knows that they have to wait to be called in. and those who are associated with the patient knows that he/she has gone to the lab and they wouldn’t disturb."(39 years old, female, caregiver)*
		Two hundred Shillings or less is the ideal cost for a POC VL test.	*“I would be willing to pay because it concerns my child’s health. If it is compulsory to pay, you have to pay because it helps you to know your child’s health progress…I think 100 shillings, 200 shillings or less would be okay. Above 100 shillings would be an extra burden.” (56 years old, female, caregiver)*
		Ensuring confidentiality when delivering VL results.	*“I think it’s important you first ask me where I am because you could be giving me the results and yet I’m in a place where I’m not able to talk to you freely.” (37 years old, female, caregiver)*
		Preferred method of delivering result is through calls or SMS. State the exact quantity of VL when delivering results.	*“I would love if the results will be communicated just like the way they text us or they call us… For a person who is interested, at least they should tell send us the text and tell us the amount of virus according to the viral load results how they came.” (39 years old, female, caregiver)*

### Perceived Positive Impacts of POC VL Testing Intervention

#### Rapid return of results or turnaround improves caretaking and facilitates timely interventions

Key informants unanimously acknowledged the benefits of a rapid return of results or turnaround time with POC VL testing. Frequently, patients/caregivers indicated that routine VL monitoring, coupled with adherence counseling, informed their health status and encouraged ART adherence by offering them an active way of receiving feedback on how their treatment and adherence are impacting their health. Yet, patients who previously received SOC, centralized testing reported experiencing long delays or misses in results delivery. Providers consistently reported the prolonged turnaround time of centralized VL testing, lasting weeks to months, as an impediment to the delivery of timely interventions, including adherence counseling, treatment adjustments, and DRM testing. In contrast, patients/caregivers who underwent POC VL testing in the Opt4Kids and Opt4Mamas studies generally reported receiving their testing results within one or two days after sample collection. Patients/caregivers were appreciative towards POC VL testing for being able to know their statuses and receive intervention quicker. This motivated patients/caregivers to reflect and make immediate improvements in ART adherence. Providers also indicated that having a fast results turnaround enabled rapid intervention for patients struggling with treatment failure or poor ART adherence.

#### Decentralization potentially reduces cost and increases the accessibility of VL testing

Providers and policymakers anticipated potential cost reduction and increased accessibility of VL testing with POC VL testing implementation over centralized VL testing. They highlighted that POC VL testing eliminates the process of transporting samples to centralized laboratories, which reduces the need for courier services and ultimately lowers testing costs. Furthermore, providers and policymakers believed that implementing POC VL testing reduces sample delivery delays for geographically remote areas that face additional challenges in transportation.

#### Increased testing frequency improves VL monitoring of children and pregnant/postpartum women

Providers explained that patients can experience drastic changes to drug adherence within the current recommended six-month VL testing interval, which may directly impede VS. Caregivers and providers articulated support for three-month testing for children because children face unique barriers to ART adherence compared to adults. For example, children are generally reliant on caregivers to maintain adherence. Many children have several caregivers simultaneously providing care for them, with each one having different approaches to administering the medications. This may result in inconsistent drug-giving or -taking behavior among children. Providers also advised three-month testing for pregnant/postpartum women because more frequent VL testing might help achieve and improve monitoring of VS to minimize the possibility of MTCT.

### Perceived Challenges with POC VL Testing Intervention

#### Acceptability of POC VL testing in the healthcare sector

Several patients reported concerns over observed discrepancies between the POC and the centralized VL testing results. Since intervention group participants could still chose to undergo centralized VL testing through their routine care providers during the study period, some who received both types of VL tests expressed skepticism towards POC VL testing after observing discrepant results. Although providers and policymakers generally acknowledged the advantages of the POC VL testing, they also articulated concerns with quality control and its validity in informing clinical decisions. Providers expressed that they are more comfortable utilizing a validated test. Thus, to build confidence, both for providers and patients, in validity of POC VL testing, providers felt that robust validation and verification processes would need to be conducted.

#### Absence of supply chain to support POC diagnostics

Providers and policymakers highlighted gaps in the supply chain of testing reagents for POC devices. A lab manager, for example, reported that functional POC machines are available in healthcare facilities but are not used because there was no channel to purchase testing cartridges after their contract ended with implementation partners. The absence of an affordable, dependable supply chain left functional devices to be underutilized.

#### Capacity limitations

Providers also raised concerns about the shortage of human resources to support POC diagnostics. Providers and policymakers saw the benefits of POC testing. They expressed the desire to leverage its multi-disease testing capability for other infectious diseases such as COVID-19, especially for hard-to-reach areas. Yet, they also worried about the struggles of prioritization and the increasing demand for staff. This was evident during the COVID-19 pandemic, where laboratory resources were reallocated to support high volumes of COVID-19 testing, causing extensive delays for VL testing. Furthermore, only a small number of designated clinical staff were trained to perform and interpret POC VL testing on POC devices that were simultaneously used for TB testing during the study period. Utilizing POC machines for multi-disease testing without capacity building, such as hiring more staff and purchasing more devices, will more likely hinder than help overcome the current barriers of the testing system in Kenya.

#### Integration of POC VL testing into the current testing guidelines

Lastly, key informants described integration issues between the POC VL testing system and routine clinic VL testing procedures. Patients who chose to continue their routine care reported feeling burdened by receiving testing from both routine clinics and POC VL testing facilities. One provider revealed confusion among healthcare staff in the clinical process due to conflicting recommendations between the national guidelines and the POC VL testing protocols, specifically in testing frequency.

#### Anxiety toward blood draws discourages patients from higher frequency VL testing

Patients/caregivers indicated some pervasive distrust and fear towards VL testing due to phlebotomy or blood draws. When asked about the negative aspects of three-month VL testing, one mother responded, "*it drains blood from the body*" and a nursing officer corroborated this sentiment that patients often say, "*they are drawing blood, you'll be anemic.*" Several of our child participants identified pain from blood draws as their primary barrier for undergoing VL testing. Aware of these sentiments, most providers also rejected the proposal of monthly VL testing, suggesting that more frequent VL testing than every three-months would discourage patients from receiving any VL testing, as its benefits outweigh the negatives of the physical and psychological distress brought on by blood draws.

### Suggested Areas of Improvement for VL Testing Scale-Up

#### Expanding the POC VL testing scheme

Since the most substantial areas for improvement for centralized VL testing were shortening the results turnaround time, many felt POC VL testing had a significant role in programmatic use for HIV treatment monitoring. Providers and policymakers advocated for continual and expanded access to POC diagnostics, with several considerations for optimization and sustainability.

#### Optimizing clinical management, workload, and utilization of POC diagnostics

Implementing a robust health information system and conducting quality checks locally can optimize clinical management, workload, and utilization of POC diagnostics. Policymakers remarked that traditional paper-based data systems have disadvantages in data duplicity, where the same data is entered into different sources by different users. Thus, this leads to data losses across the cascade, as providers experience added workload, therefore, see less value in entering the same data across multiple points. Providers echoed this by reporting that gaps within health information sharing, especially with notifying the release of patient VL results, is an important factor that led to missed VL results discussion with patients and further intervention for non-suppression. On the other hand, electronic records system provides a centralized platform to store health data, which reduces data duplicity and allows data sharing on the national level. Thus, benefits were seen with using an electronic records system for the scale up of VL testing to assure clinical care continuity, health information sharing, and reduction of workload for providers.

Concerns with quality control led some providers to be less willing to utilize POC VL testing. Providers felt like they needed more validation and control over the process in order to be able to rely on POC VL testing. Among providers and policymakers, there was consensus to conducting quality checks locally at the facilities where POC VL testing is implemented to boost clinical staff confidence.

#### Capacity building and partnership development for sustainable VL testing system

Policymakers and providers believed that a sustainable VL testing system will require capacity building and collaborative partnerships. With considerations for the increased workload brought by POC diagnostic implementation, providers and policymakers recommended expanding testing capacity by training more staff and equipping labs with POC testing devices. In addition, policymakers advised building collaborative partnerships between key stakeholders involved in implementing POC VL diagnostic as a vital step in developing a seamless process for VL testing procedures and patient management. The integration of POC VL testing into the current centralized system involves streamlining testing recommendations and the development of new guidelines. This process requires effective communication between different stakeholders.

#### Envisioning the ideal testing experience

Key informants were asked to envision the ideal testing experience by providing recommendations for the optimal testing frequency, blood drawing amount, testing setting, testing cost, and results delivery method. Despite reported anxiety experienced during blood draws, there was a clear consensus that every three months was the ideal testing frequency for children and pregnant/postpartum women among all groups of key informants. Patients/caregivers reported that receiving sufficient explanations and education from providers could successfully alleviate their concerns with blood drawing. However, some providers and patients/caregivers also suggested modifying the sampling amount to small amounts or even finger pricks, if feasible, to relieve patients' anxiety during the procedure. When asked about the ideal setting for performing VL testing, providers generally preferred VL testing to be conducted at the healthcare facility's laboratory, by a lab technician instead of directly by themselves, to assure the quality of testing, minimize contamination, improve clinic flow and prevent increasing their workload. Surprisingly, most informants, including patients/caregivers, indicated they were willing to contribute towards VL testing costs. Most patients/caregivers determined 200 Kenyan Shillings (approximately USD 1.85) or less to be a reasonable price for paying out-of-pocket for a POC VL test for which they could receive the result before leaving the facility.

In addition, patients/caregivers desired punctual and informative communication for VL testing results. Overwhelmingly, patients/caregivers reported phone calls or text messaging as the preferred method of results communication. Patients/caregivers said that delivering results by phone can save trips to the clinic and ensure timely reception of results. However, participants also raised concerns about inadvertent disclosure of HIV status, as phone sharing between family and community members is a common practice in Kenya. To ensure the confidentiality of medical information, several patients/caregivers proposed to confirm the patient's identity before delivering VL results. Patients/caregivers recommended the content of the call or text message to include the patient's name, followed by the exact number of HIV-1 RNA copies detected from testing. Other patients/caregivers also suggested incorporating words of encouragement to motivate ART adherence.

## Discussion

This study contributes to the limited literature that qualitatively investigates the experiences of POC VL testing in a resource-limited setting, with a focus on children and pregnant/postpartum women, and elicits approaches to optimization and sustainability from provider and policymaker perspectives. We report overwhelmingly positive perceptions of POC VL testing, with our key informants regarding it as more accessible and timelier as compared to centralized VL testing. Nevertheless, our key informants also identified several challenges to realizing the full benefits of POC VL testing, and pointed out areas of improvement in quality control, human resource and infrastructure capacity, supply chain management, and POC and centralized VL testing system integration. Overall, there appears to be enthusiasm for the scale-up of POC VL testing and timely return of results from patients/caregivers, providers, and policymakers in Kenya. The key questions now center on how the implementation of POC VL testing alongside centralized VL testing truly impact clinical outcomes, and if deemed beneficial, how to develop an optimal and sustainable centralized and POC VL testing program.

POC VL diagnostics can maximize benefits received from routine VL monitoring by addressing critical challenges with centralized VL testing. The long results turnaround time of centralized VL testing, even if down to < 14 days now, diminishes some of the benefits of VL monitoring by not having rapid return of results and the ability to counsel and make clinical decisions in a more timely fashion [9, 31]. The decentralized approach of POC VL testing, on the other hand, saves the time spent on transportation, facilitating prompt delivery of counseling to motivate ART adherence and opportune switch of ART regimen for those with treatment failure [32–35]. In addition, some POC VL testing devices are portable, which increases the accessibility of VL testing in hard-to-reach areas [36]. The decentralized, more accessible model of POC testing has great potential in complementing centralized VL testing, or for multi-disease testing, in Kenya to fill gaps or needs where they currently exist. Ultimately, while the reduction in turnaround time is a key element of POC VL testing—and patients/caregivers, providers, and policymakers were clearly most enthused about this aspect of POC VL testing in our study, it remains unclear how such reductions in turnaround time truly translate to improved clinical outcomes, as the studies to date have mixed findings. For instance, in the Opt4Kids and Opt4Mamas studies, the intervention groups who underwent more frequent POC VL testing, alongside other interventions, did not have improved VS compared to the control groups undergoing SOC [37, 38]. Similarly, another study in South Africa with pregnant/postpartum women also did not find any significant improvements in VS [39]. On the other hand, one study in South Africa that coupled POC VL testing with task shifting showed a higher VS rate and retention in HIV care in the intervention group [40], and another study in Uganda showed POC VL testing resulted in higher one-year VS rate post-intervention [40, 41].

With the versatility of POC diagnostics, there has been demand to leverage the technology for multi-disease testing in resource-limited settings. However, the feasibility and operational challenges of integrating POC diagnostic for multi-disease testing remains unclear. In our study, key informants identified human resource capacity and prioritization as the main considerations for multi-disease testing. A study done in Zimbabwe that examined multi-disease testing for HIV and TB on GeneXpert® implemented a differentiated care model that prioritized testing for key populations at higher risk of adverse outcomes. This effectively minimized disruption to POC TB and HIV testing while maximizing the utility of the devices [42]. Nonetheless, there are other considerations for POC multi-disease testing identified by the WHO, many of which are parallel to those proposed in this study, such as coordinated planning, developing standard operating procedures, quality management and validation, supply chain and inventory management [43]. Further research is needed to assess the prospects of POC multi-disease testing in improving health outcomes and challenges that may arise.

Decentralization brought by POC VL testing increases the complexity of quality control and supply chain management procedures. As POC VL testing shifts sample processing outside of centralized laboratories, it adds uncertainty to testing quality and accuracy. It has been proposed that establishing country-owned external quality assessment (EQA) programs can help address quality issues to ensure diagnostic accuracy [44]. This arose as a finding in our work, where informants indicated that building confidence for the locally operated POC VL testing required the validation, verification, and EQA checks to be led and conducted locally. In addition, the successful establishment, supervision, and maintenance of a long-term reagent supply chain may be more difficult to achieve under a decentralized system, as reagents needed for POC VL testing devices have to be transported to different locations, including hard-to-reach health facilities. Arguably, supply chain issues apply to all types of medical commodities so these issues should be solvable. Yet, there is a paucity of data to support ways to optimize the supply chain management for POC diagnostics in low and middle income countries (LMIC) [45]. Thus, further research is needed to address ways to optimize supply chain management for POC diagnostics.

Our key informants also highlighted that POC VL testing implementation, especially if implemented at a higher frequency for certain subpopulations, might worsen existing challenges, such as increasing clinical flow or burden. Our key informants indicated that capacity building and transitioning to an electronic records system could reduce the workload for providers by minimizing data duplicity. A study performed in Kenya found that task-shifting ART delivery from clinical staff to community care coordinators ensured similar health outcomes for clinically stable patients living with HIV and reduced the number of clinical visits by half [46]. Another recent study performed in South Africa found that POC VL testing enabled early task-shifting of the caregiving responsibility of VS patients from professional nurses to a lower cadre of nurses acceptably and feasibly [32, 40]. A similar model of task-shifting, plus developing a robust health information system, may be adapted for the future scale-up of POC VL testing in Kenya to mitigate human resource barriers. A key consideration in the scale-up of POC VL testing will be to do so in a manner that least burdens the already overburdened staff at the facilities in LMICs.

Furthermore, our key informants recommended streamlining POC VL testing into the existing health system operations, some of which are already in process in Kenya [47]. Developing good partnerships between key stakeholders is a necessary step to the integration of the two testing methods. In addition, it is essential to define the role that POC VL testing will play in the HIV treatment cascade and develop clear action plans to guide clinical decision-making and patient management [35]. Given some of the advantages of POC VL testing, such as decentralization and portability, it has been proposed that such technology could be especially fruitful in enhancing patient outcomes for vulnerable and hard-to-reach populations [16].

Aside from anticipating some of the above implementation barriers, we also note key considerations for the optimalization of VL testing under a resource-limited environment with unique sociocultural context. These factors would be essential to consider in the scale-up of POC VL testing but are also applicable to centralized VL testing. For example, patients/caregivers in our study reported 200 Kenyan Shillings (approximately $1.85) or less, admittedly only a fraction of the estimated $25 to $30 cost per test, to be a reasonable expense they would be willing to pay out-of-pocket for POC VL testing [21, 22]. The fact that patients/caregivers were willing to pay money for the possibility of receiving results sooner signals the value of POC VL testing to them. Therefore, regardless of centralized vs. POC testing component, better systems need to be built within VL results reporting within resource-limited settings that helps address the desire that patients/caregivers themselves have for getting this data back to them soon. We also found that anxiety towards phlebotomy may serve as a critical barrier that discourages patients from VL testing, especially among children. Other factors such as stigma, cultural beliefs, and previous adverse experiences can all heighten fear towards phlebotomy and deter patients from receiving timely VL testing even if the health system were ready to offer it to the patient [48–51]. When designing education programs to overcome such barriers, it is crucial to address all the different social, cultural, and experiential factors contributing to patients' testing decisions.

Lastly, our study in one of the few that assesses the acceptability of higher-frequency testing. We found that three-month testing frequency was acceptable, and even desirable, for many patients/caregivers, providers, and policymakers, articulating the limitations of six-month testing in delaying timely clinical interventions for particularly vulnerable subpopulations such as children and pregnant/postpartum women. A study from Uganda simulated the efficacy of different testing frequencies in detecting treatment failure, noting that all schemes of shorter frequencies of testing as compared to testing intervals of six months resulted in higher cases of previously undetected treatment failure [50]. Our data from the Opt4Kids study suggests, for instance, that higher frequency VL testing in children does detect more episodes of viremia, and the rates of HIV drug resistance mutations are alarmingly high (100% of all tested samples had any mutation, and 85% had at least one major mutation) among these children, a third-of whom required an ART regimen change [37, 52, 53]. Thus, more frequent VL testing may result in greater detection of treatment failure and, ideally, more optimal management of such individuals. However, testing frequency must be balanced with costs and other local considerations.

One of the most significant strengths of our qualitative study is that it provided diverse insights to POC VL testing by incorporating the perspectives of patients/caregivers, providers, and health system policymakers, and has a high potential for transferability to other LMICs considering optimal scale-up of VL testing programs. However, our study also has several limitations. We were unsuccessful in eliciting meaningful interviews with our adolescent participants and chose to terminate them sooner than the desired number of interviews. Additionally, a coding and analysis effort driven exclusively by Kenya-based team members may have resulted in different conclusions or emphases on particular findings, despite the fact that the Seattle-based coding and analysis team met with the Kenya-based team on many occasions. Lastly, our study was conducted during the global COVID-19 pandemic, which may influence our findings, for example, the convenience of saving trips to clinics during the COVID-19 pandemic may influence participants to favor POC VL testing implemented in the study over the SOC centralized VL testing process. The pandemic may also add weight to certain themes more than others (e.g., supply chain shortages), though we anticipate such themes would have been identified regardless of the pandemic.

## Conclusion

We found that POC VL testing, coupled with higher frequency testing, is deemed highly desirable, especially for vulnerable subpopulations such as children and pregnant/postpartum women living with HIV, by various groups of individuals, from patients/caregivers, providers, and policymakers in Kenya. The more accessible, decentralized model of POC VL testing was felt to be able to overcome challenges currently facing centralized VL testing. However, several areas of improvement including quality control, human resource and infrastructure capacity, supply chain management, and integration of VL testing systems, were delineated, with accompanying potential solutions for larger scale-up of POC VL testing. Further investigations are needed to identify methods to achieve optimized and sustainable POC VL testing in LMICs.

## Supplementary Information

**Additional file 1. **Opt4Kids In-depth Interview Guide (for caregivers of study participants and adolescent participants ages 13-14).

**Additional file 2. **Opt4Kids & Opt4Mamas Key Informant Interview Guide (for various providers and policymakers).

## Data Availability

The dataset generated and analyzed during the current study are not publicly available in accordance with our ethics reviews. The corresponding author will allow sharing of de-identified data, data codebook, and other data elements upon request and ethics approval, and upon submission of relevant documents, such as research protocol, and signed data access agreement.

## Abbreviations

ANC: Antenatal care
ART: Antiretroviral treatment
CLHIV: Children living with HIV
COVID-19: Coronavirus disease 2019
DRM: Drug resistance mutation
EQA: External quality assessment
KII: Key informant interviews
LMIC: Low- and middle-income countries
MTCT: Mother-to-child Transmission of HIV
POC: Point-of-care
SOC: Standard-of-care
TB: Tuberculosis
VL: Viral load
VS: Viral suppression
WHO: World Health Organization
