# Supplementary material for: “After viral load testing, I get my results so I get to know which path my life is taking me”: qualitative insights on routine centralized and point-of-care viral load testing in western Kenya from the Opt4Kids and Opt4Mamas studies
Source: BMC Health Serv Res. 2022 Dec 17;22:1540. doi: 10.1186/s12913-022-08593-z (PMC9758673; doi:10.1186/s12913-022-08593-z)
Supplement: Supplementary file 1 — Additional file 1. Opt4Kids In-depth Interview Guide (for caregivers of study participants and adolescent participants ages 13-14). [file 12913_2022_8593_MOESM1_ESM.pdf]

Opt4Kids In-depth Interview Guide (for caregivers of study participants and adolescent participants ages 13-14)

Note: The questions suggested below are simply guides; you are not compelled to ask all of the questions below. Prompts are meant as suggestions only, not as a checklist. Use prompts to engage participant in further discussion. If asking questions of a caregiver, all questions pertain to their child participating in the study and not themselves unless otherwise indicated.

"Thank you for agreeing to meet with me today. I really appreciate your time. My name is [name] and I am a [role] with the Opt4Kids study. We are conducting some interviewers with our study participants and their caregivers to better understand a few things."

Required: "May I read you the informed consent form now? [After reading the form] What questions do you have? [Answer all questions] OK go ahead and sign the form. Let's begin now and I will start the recording."

A. General Barriers to Taking ART

Establishing rapport

- How old are you (or your child participating in the study)?
- When were you (or your child participating in the study) first diagnosed with HIV? How was that experience? Do you remember what some of your initial reactions were like?
- When did you (or your child participating in the study) first start taking any ARVs?

How has your experience (or your experiencing giving the child ARVs) been so far with ARVs? How about others who you know are also taking ARVs?

- What are some positive aspects of taking ARVs?
- What are some negative aspects? What do you wish would be different?

Barriers to ART (Focus on 5 broad categories)

- What problems do people in your community generally face when taking ARVs?
    - ***individual-*** adherence, acquired HIV drug resistance, drug-drug interactions
    - ***interpersonal-*** disclosure, stigma, family/partner support, health/HIV status of other children, provider attitudes/training
    - ***organizational-*** provider strikes, drug stock outs, transportation to facilities, costs of coming to facilities/missing work, delay in feedback of testing
    - ***societal/cultural norms-*** stigma
    - ***policy/structural-*** *discrimination against ethnic minorities, poverty*
- What problems do children/adolescents generally face when taking ARVs? How are some of these different from barriers facing adults?
- What problems have you (or your child participating in the study) encountered so far in taking ARVs?
- How has the novel coronavirus 2019 (COVID-19) pandemic impacted your (or your child participating in this study) overall well-being? How has COVID-19 impacted your HIV care and ability to take your ARVs?

Facilitators for ART use (Focus on 5 broad categories)

- What things help people take their ARVs in your community?
- What things help children take their ARVs?
- What things help you take your ARVs (or your child participating in the study)?

B. VL Literacy and Experience with VL & DRM Testing in Routine Care

Can you explain to me what you understand about the HIV term "viral load"? What does it mean to you?

- How have others explained this term to you?
- Why do your providers want to monitor your viral load?
- How is the viral load used to guide your HIV treatment?
- What does "high" or "low" viral load mean? What does "supressed" mean?
- What is your understanding about how often your (child's) viral load should be checked?
- What more would you like to learn about viral load? What more can providers explain to you about viral load?

Have you (or your child participating in the study) had any VL tests done so far outside of the Opt4Kids study? What has your experience been like so far with VL testing, if any? Positive or negative? Explain why?

- How many times have you had your viral load checked so far?
- How much time passed generally between your child having their blood taken and the result being communicated back to you?
- Who has been returning the results to you? And how?
- Did your provider discuss the VL test result with you? If so, what did they say?
    - How did you react to what the provider said?
- What would you like to see done differently about providers giving you your child's VL results?
- Would you ever feel comfortable telling a provider that you want a VL checked for you (your child)?

"If providers are worried that the HIV medications you are taking are no longer working against your virus, they may get special testing done called drug resistance testing."

What has your experience been like so far with drug resistance testing, if any?
Have you (your child) had any DRT tests done so far?

- How much time passed between having your blood taken and the result being communicated back to you?
- Did your provider discuss the DRT test result? If so, what did they say? How did you react to what the provider said?
- What would you like to see done differently about providers giving you your (child's) DRM results?

C. Experience with POC VL & DRM Testing (for POC group only)

As you know, your child is undergoing point-of-care viral load testing every 3 months at this facility.

What has your experience been like so far with the use of POC VL testing?

- Time to return of results, timeliness
- Frequency of testing
- Communication to you by study staff
- Communication from facility staff/providers

What are some positives aspects of the POC testing? What are some negative aspects?

How did the study team communicate the results of the POC VL testing to you?
How would you improve communication about your child's viral load result for the future?

Did your provider discuss any of the POC VL test results with you?

- If so, how did they give you the result? By phone, in-person?
- What did the provider say to you? What was the content of the counseling message you heard?
- What did you like about the provider communication or counseling, or not like about it?
- What would you like to see changed about provider communication or counseling in the future?

What types of results have you received so far? E.g. LDL, high, suppressed or not suppressed?
Did learning your POC VL test results change anything you did?

- Did it change any aspect of you taking your ARVs? Do you know if you/your child undergo any additional testing based on the VL results (e.g. drug resistance testing)?
- Did it motivate you to keep taking your ARVs as you were? Or change or improve the way you were taking your ARVs?

What would be the best place for getting a VL test? Example, in the clinic room while you are seeing the provider? In the waiting areas? In lab?

Ideally, how often do you think your (or your child's) viral load should be checked? Is 3 months too often? Would your ideal be every 3, 6, or 12 months?

- How concerned are you about frequent blood draws? What are your concerns about the frequent blood draws? How would these concerns depend on the amount of blood being drawn? Would you be less concerned if we drew 1 tablespoon or blood? Would it change if we drew 1 teaspoon? How about a finger prick?

How would you like the results of viral load tests returned to you? By phone call/text, in-person? By whom? What type of information do you need/want in the messages that these persons give you? Who do you want communicating the results back to you?

Would you ever be willing to pay to have your (or your child's) VL checked? If so, how much might you be willing to pay for it?

How has the novel coronavirus 2019 (COVID-19) pandemic impacted your access to VL testing within the Opt studies?

Have you had any drug resistance testing done so far?
If yes:

- How much time passed between having your blood taken and the result being communicated back to you?
- Did your provider discuss the DRM test result? If so, what did they say? How did you react to what the provider said?
- What would you like to see done differently about providers giving you your (child's) DRM results?

- How has the novel coronavirus 2019 (COVID-19) pandemic impacted your access to DRT testing within the Opt studies?

“Is there anything else you would like to add? Again, we greatly appreciate all your time and help.”
