# Supplementary material for: “After viral load testing, I get my results so I get to know which path my life is taking me”: qualitative insights on routine centralized and point-of-care viral load testing in western Kenya from the Opt4Kids and Opt4Mamas studies
Source: BMC Health Serv Res. 2022 Dec 17;22:1540. doi: 10.1186/s12913-022-08593-z (PMC9758673; doi:10.1186/s12913-022-08593-z)
Supplement: Supplementary file 2 — Additional file 2. Opt4Kids & Opt4Mamas Key Informant Interview Guide (for various providers and policymakers). [file 12913_2022_8593_MOESM2_ESM.pdf]

Opt4Kids & Opt4Mamas Key Informant Interview Guide (for various providers and policymakers)

Note: The questions suggested below are simply guides; you are not compelled to ask all of the questions below. Prompts are meant as suggestions only, not as a checklist. Use prompts to engage participant in further discussion.

Required: "May I read you the informed consent form now? [After reading the form] What questions do you have? [Answer all questions] OK go ahead and sign the form. Let's begin now and I will start the recording."

A. General Barriers to Taking ART

Establishing rapport

- What is your occupation?
- How do you interact with HIV positive individuals? And children living with HIV? And how about pregnant women with HIV?
- How long have you been caring for children with HIV? How long have you been caring for pregnant women with HIV? What other patients do you care for?

Barriers to ART

- What barriers do people generally face when taking ARVs? (Focus on 5 broad categories)
  - ***individual-*** adherence, acquired HIV drug resistance, drug-drug interactions
  - ***interpersonal-*** disclosure, stigma, family/partner support, health/HIV status of other children, provider attitudes/training
  - ***organizational-*** provider strikes, drug stock outs, transportation to facilities, costs of coming to facilities/missing work, delay in feedback of testing
  - ***societal/cultural norms-*** stigma
  - ***policy/structural-*** *discrimination against ethnic minorities, poverty*
- What specific barriers do children face when taking ARVs? Are those any different from adults?
- What specific barriers do pregnant women face? Do these barriers change after delivery, during the 2 years after delivery?
- How has the novel coronavirus 2019 (COVID-19) pandemic impacted patient's overall well-being? How has COVID-19 impacted HIV care or ART processes at your facility (or region/counry)?

Facilitators for ART use

- What factors facilitate use of ARVs generally?
- What specific factors facilitate use of ARVs for children? Are those any different from adults?
- What specific factors facilitate use of ARVs for pregnant women? Do these factors change after delivery, during the 2 years after delivery?

B. Experience with SOC VL & DRT Testing in Routine Care

Can you explain to me what you understand about the HIV term "viral load"? What does it mean to you? How have others explained this term to you?

- How do you tend to explain this term to others? To clients? To children?

What has your experience been like so far with routine VL testing in your facility (not POC VL testing), if any?

- What do you know about the nationally recommended algorithm?
- What are your thoughts about how feasible that algorithm is? For children? For pregnant women? Is there a difference between children and adolescents?
- What are the major challenges in implementing that algorithm?
- What are your opinions about the frequency of recommended VL testing? For children/adolescents? For pregnant/postpartum women?
- How do patients learn about their VL test results?
- What, if any, education needs to occur around VL testing for patients? How do you/your clinic accomplish that? Specifically, what do you tell a patient/caregiver if VL is undetectable? Low level viremia (i.e. VL >400 but <1000 copies/mL)? Over 1000 copies/mL?
- Have you ever been trained on how to provide viral load education or counseling?

If clinician, have you ordered any VL tests so far? (*Reminder, this is in regards to routine VL testing or other VL testing through SOC approach)*

- How easy was it to order it? On a scale for 0-10 (10 being the easiest to order)
- What was the turn around time?
- How were the results reported back to you?
- How did you communicate those results back to the patient?
- What would you want to see done differently going forward?
- How has the novel coronavirus 2019 (COVID-19) pandemic impacted SOC VL testing for your patients?

What has your experience been like so far with HIV drug resistance testing, if any?
If you or your facility has requested a DRT tests done so far:

- How much time passed between having the blood taken and the result being communicated back to you?
- How were the results communicated to you? And who communicated them to you?
- How comfortable are you interpreting DRT results?
- How comfortable are you making management decisions by yourself using DRT results?
- How are DRT results usually handled at your facility? (e.g. provider reviews alone and makes decision, multidisciplinary team meeting held and group decision made, discuss with technical advisor or medical officer)
- Did you or any other provider give the patient/caregiver any education or counseling about the DRT result? If so, what did you say? How did the patient react to that?
- What would you like to see done differently about about how providers receive patient DRT results and make clinical management decisions? Would you like more training on how to interpret and use DRT results? Would you like an algorithm or other recommendation guide on how to use DRTresults?

C. Experience with POC VL & DRT Testing (if facility staff)
What has your experience been like so far with the use of POC VL testing?

- Time to return of results, timeliness
- Frequency of testing
- Communication to you by study staff

What are some positives aspects of the POC testing?
What are some negative aspects?

How did the study team communicate the results of the POC VL testing to you for a child?
How would you improve that for the future?

Did you discuss any of the POC VL test results with your patients?

- If so, how did you do it?
- What did you like about it, or not like about it?
- What would you like to see changed about it in the future?

Did learning your patients' POC VL test results change anything you did with them?

- If so, what did you change?
- Any changes to adherence counseling? To ART?

How has the COVID-19 pandemic impacted POC VL testing within the Opt studies?

What recommendations do you have on improving the use of POC VL testing at your facility, for your patients?

- What would be your ideal setting for getting a VL test?
    - Example, in the clinic room while you are seeing the provider? In the waiting areas?
- Who should run the test?
- Would you feel comfortable running the test?
- How often should POC VL testing be done? For children/adolescents? For pregnant and postpartum women? Is monthly during pregnancy or postpartum too often?

What has your experience been like so far with HIV drug resistance testing within the Opt studies?

- How much time passed between having the blood taken and the result being communicated back to you?
- How were the implications of the results communicated to you? And who communicated them to you?
- Did you or any other provider discuss the DRT test results with the patient? If so, what did you/that provider discuss with the patient? How did the patient react to that discussion?
- What would you like to see done differently about providers discussing DRT results with patients?
- How has the COVID-19 pandemic impacted DRT testing within the Opt studies?
